# Drug-coated balloon versus conventional balloon angioplasty of hemodialysis arteriovenous fistula or graft: A systematic review and meta-analysis of randomized controlled trials

**DOI:** 10.1371/journal.pone.0231463

**Published:** 2020-04-14

**Authors:** Min-Tsun Liao, Meng-Kan Chen, Mu-Yang Hsieh, Nai-Lun Yeh, Kuo-Liong Chien, Chih-Ching Lin, Chih-Cheng Wu, Wei-Chu Chie

**Affiliations:** 1 Division of Cardiology, Department of Internal Medicine, National Taiwan University Hospital Hsinchu Branch, Hsinchu, Taiwan; 2 Department of Family Medicine, National Taiwan University Hospital Hsinchu Branch, Hsinchu, Taiwan; 3 College of Medicine, National Taiwan University, Taipei, Taiwan; 4 Department of Internal Medicine, National Taiwan University Hospital, Taipei, Taiwan; 5 Institute of Epidemiology and Preventive Medicine, College of Public Health, National Taiwan University, Taipei, Taiwan; 6 Division of Nephrology, Department of Medicine, Taipei Veterans General Hospital, Taipei, Taiwan; 7 School of Medicine, National Yang Ming University, Taipei, Taiwan; 8 Institute of Biomedical Engineering, National Tsing-Hua University, Hsinchu, Taiwan; 9 Cardiovascular Center, National Taiwan University Hospital Hsinchu Branch, Hsinchu, Taiwan; 10 Institute of Cellular and System Medicine, National Health Research Institute, Zhunan, Taiwan; 11 Department of Family Medicine, National Taiwan University Hospital, Taipei, Taiwan; University of Wisconsin, UNITED STATES

## Abstract

**Background:**

Restenosis remains a significant problem in endovascular therapy for hemodialysis vascular access. Drug-coated balloon (DCB) angioplasty decreases restenosis in peripheral and coronary artery diseases. The aim of this systematic review and meta-analysis is to assess the patency outcomes following DCB angioplasty, as compared to conventional balloon (CB) angioplasty for the stenosis of hemodialysis vascular access.

**Methods:**

A comprehensive search in the MEDLINE, EMBASE, and CENTRAL databases was conducted in order to identify eligible randomized controlled trials evaluating DCB angioplasty for hemodialysis vascular access dysfunction. The primary endpoint was the 6-month target lesion primary patency and the secondary endpoints were 12-month target lesion primary patency and procedure-related complications. Risk ratios (RR) were pooled and relevant subgroups were analyzed separately.

**Results:**

Eleven randomized controlled trials comprised of 487 patients treated with DCB angioplasty and 489 patients treated with CB angioplasty were included. There were no significant differences in the target lesion primary patency at 6 months [RR, 0.75; 95% confidence interval (CI), 0.56, 1.01; *p* = 0.06] and at 12 months (RR 0.89; 95% CI, 0.79, 1.00; *p* = 0.06). The absence of benefit for the DCB group remained, even in the arteriovenous fistula subgroup or the subgroup of studies excluding central vein stenosis. The risk of procedure-related complication did not differ between the two groups (RR 1.00; 95% CI 0.98, 1.02; *p* = 0.95).

**Conclusion:**

DCB angioplasty did not demonstrate significant patency benefit for the treatment of hemodialysis vascular access dysfunction. Wide variations in patency outcomes across studies were noted. Further studies focusing on specific types of access or lesions are warranted to clarify the value of DCB for hemodialysis vascular access. (PROSPERO Number CRD42019119938)

## Introduction

Hemodialysis vascular access dysfunction is one of the major causes of morbidity and mortality in patients with end-stage renal disease (ESRD). The most common cause of vascular access dysfunction is venous stenosis caused by neointimal hyperplasia. Percutaneous transluminal angioplasty (PTA) is increasingly used as the primary therapy for vascular access dysfunction. Although it is fast and convenient, the primary patency rate of conventional balloon (CB) angioplasty remains relatively low, ranging from 26–58% at one year [1, 2]. A variety of strategies have been explored, such as cutting balloon, high-pressure balloon, and covered stents, to improve the durability of therapy. Nonetheless, there is a constant need for repeat interventions, which remains a substantial burden on the healthcare system.

The predominant pathology of a venous stenosis is neointimal hyperplasia, which is characterized by rapid smooth muscle proliferation. Balloon angioplasty, a procedure used to treat stenosis, creates deep fractures into the neointimal tissues by forceful intraluminal dilatation. The procedure may incite various degrees of proliferative response at the site of balloon dilatation. Paclitaxel prevents neointimal hyperplasia by causing cellular apoptosis and inhibiting smooth muscle cell migration. The use of paclitaxel-coated angioplasty balloons has been reported to decrease restenosis in patients with femoropopliteal artery stenosis [3] and coronary in-stent restenosis [4]. The effects of drug-coated balloons (DCB) on hemodialysis vascular access were recently explored in various randomized studies. Nonetheless, the sample sizes of these studies were usually small and conflicting findings were reported in some of these studies.

It is the aim of this systematic review and meta-analysis to comprehensively review the most up-to-date randomized controlled studies and to compare the patency outcomes of DCB against CB angioplasty for the treatment of hemodialysis vascular access dysfunction.

## Methods

### Search strategy

This systematic review and meta-analysis was designed according to the principles set by the Preferred Reporting Items for Systematic Reviews and Meta-analysis (PRISMA) checklist [5]. We conducted a comprehensive literature search in PubMed, EMBASE, and Cochrane Controlled Register of Trials (CENTRAL) from database inception to Mar 9, 2020, using the terms (‘Arteriovenous Graft’ OR ‘Arteriovenous Fistula’ OR ‘Arteriovenous Shunt’ OR ‘Vascular access’ OR ‘Dialysis access’ OR ‘Arteriovenous Anastomosis’ OR ‘Arteriovenous Shunt, Surgical’ OR ‘Blood Vessel Prosthesis’) AND (‘Drug Eluting Balloon’ OR ‘Paclitaxel Coated Balloon’ OR ‘Drug Coated Balloon’ OR ‘Paclitaxel’). The detail of the search strategy is listed in **S1 File**. The language of the publications was limited to English and the studies were limited to human trials. We also hand-searched the references from original and review articles for additional studies.

Two independent investigators (Min-Tsun Liao and Meng-Kan Chen) searched and reviewed all identified studies. The objectives, methodology, and inclusion criteria for study enrollment were prespecified. The protocol of the study was registered in the International Prospective Register of Systematic Reviews (PROSPERO) (Number CRD42019119938). The inclusion criteria include: (1) hemodialysis patients with stenotic arteriovenous fistula (AVF) or graft (AVG), (2) head-to-head comparison between DCB angioplasty and CB angioplasty, (3) primary patency at 6 months and 12 months, and (4) randomized controlled trials (RCTs). Titles and abstracts were used for reviewing the relevance of a study. If a study met the inclusion criteria, the full manuscript would then be reviewed. Conflicts between two reviewers were discussed through the consensus of a third reviewer (Nai-Lun Yeh). Final quantitative synthesis of patency outcome was analyzed.

### Data extraction

Study designs and setting, publication year, type of balloon, stenosis assessment of enrollment and follow-up, and clinical outcomes including primary patency of target lesion and access circuit at 6 and 12 months and complications were extracted from selected RCTs. All authors of the included studies were contacted for more detailed information, but only one author kindly provided us additional data.

### Quality assessment

We assessed the quality and risk of bias of the included studies using the domains of the Cochrane Collaboration tool [6]. A judgment of “high,” “unclear,” or “low” risk of bias was provided for each included study, including random sequence generation (selection bias), allocation concealment (selection bias), blinding of participants and personnel (performance bias), blinding of outcome assessment (detection bias), incomplete outcome data (attrition bias), and selective reporting (reporting bias).

### Statistical analysis

We used weighted relative risk with random effect and 95% confidence interval for the pooled estimates of the dichotomous outcomes. To assess the heterogeneity across studies, I^2^ statistic was used to determine the variance across studies. Results were considered statistically significant if the P value was less than 0.05. Deeks’ funnel plot asymmetry test was performed to evaluate the publication bias [7]. We also analyzed the subgroups including the AVF-only subgroup, the non-central venous stenosis (CVS) subgroup, and the AVF-only and non-CVS subgroup. Statistical analyses were performed with Review Manager (RevMan) version 5.3 (Copenhagen: The Nordic Cochrane Centre, The Cochrane Collaboration, 2014).

## Results

### Study selections

**Fig 1:**
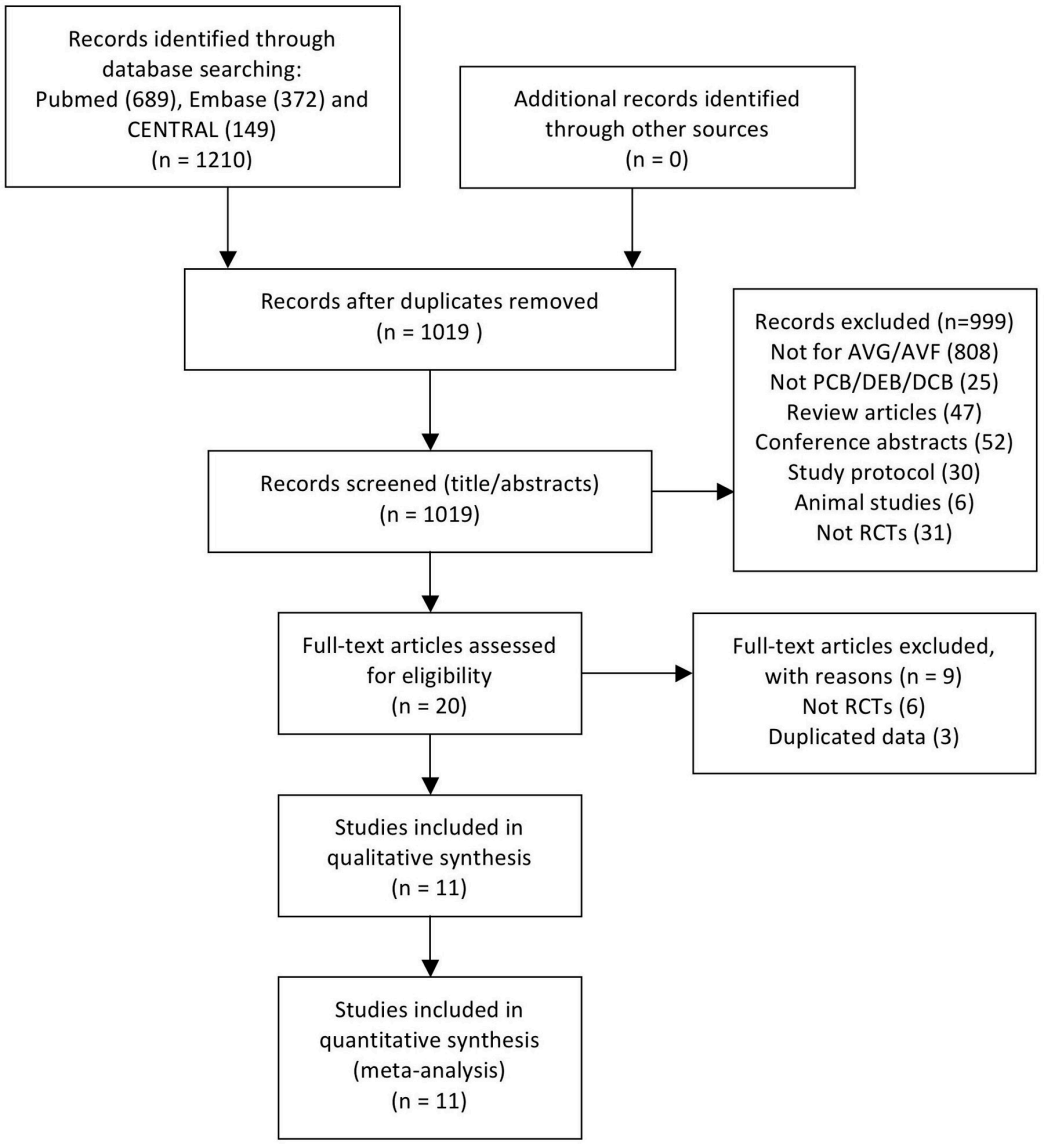
Flow chart for the selection of studies. Abbreviations: AVG, arteriovenous graft; AVF, arteriovenous fistula; PCB, paclitaxel-coated balloon; DEB, drug-eluting balloon; DCB, drug-coated balloon; RCT, randomized controlled study.

We obtained a total of 1210 publications from the initial search and 191 publications were duplications. After reviewing the titles and abstracts, 999 publications were excluded. After reviewing the full text of 20 publications, 11 publications were included in the final qualitative and quantitative analyses, with a total of 976 patients [8–18]. The flow diagram of the systematic search process is depicted in **Fig 1**.

### Assessment of methodologic quality

**Fig 2:**
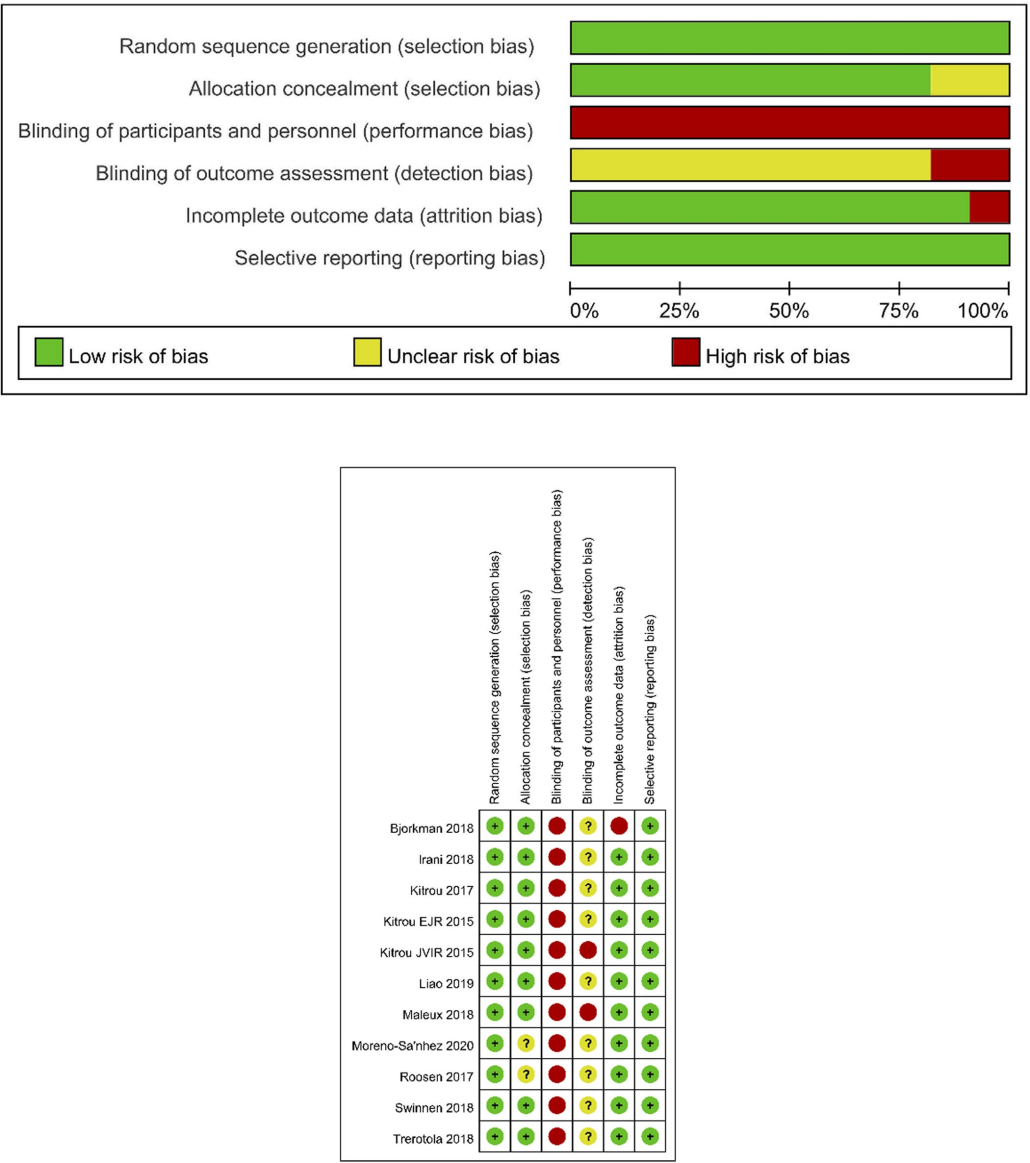
Risk of bias assessments for included studies A. Risk of bias graph B. Risk of bias summary.

The quality of randomized controlled trials was assessed using the Cochrane risk-of-bias tool. All studies were evaluated in the following seven domains: random sequence generation (selection bias), allocation concealment (selection bias), blinding of participants and personnel (performance bias), blinding of outcome assessment (detection bias), incomplete outcome data (attribution bias), selective reporting (reporting bias), and other biases. The quality in each domain was assessed as high, low, or unrecognized risk. The different types of biases included 2 study with unclear allocation bias, 1 study with high-risk attrition bias, 2 studies with high-risk detection bias, 9 studies with unclear detection bias, and 11 studies with performance bias. Only 4 studies had prespecified study protocols. The details of the quality assessment were provided in **Fig 2**.

### Summary of studies

The characteristics of the included studies are summarized in **Table 1**. Two studies were sponsored by manufacturers and other studies were initiated by investigators. There were 5 studies with a multi-center design. 3 studies were non-blinded while the others were single-blinded. The control arm in 6 studies was the CB, while the control arm in the other 5 studies was the high-pressure balloon. There were 5 studies that only enrolled patients with AVFs, one study only enrolled patients with AVG, and the other 5 studies enrolled patients who have either AVFs or AVGs. One study only enrolled patients with central venous stenosis, while 5 studies excluded those with central venous stenosis. Dialysis access thrombosis were excluded in 7 studies. Target lesion patency was the primary outcome in 10 of the 11 studies. The follow-up protocol varied widely between these studies, including clinical assessment, ultrasonography, and angiography. The characteristics of patients and outcomes in each of the eligible RCTs are summarized in **Table 2**. The type of vascular access in the 11 studies were mainly AVFs (849 cases, 87%).

**Table 1:** Characteristics of eligible RCTs analyzing the DCB and CB angioplasty in hemodialysis vascular access.

Study (Year)	Designs & Setting	Type of Balloon		Stenosis Assessment at Enrollment	Stenosis Assessment at Follow-up	Primary Endpoint
		DCB	Control			
Kitrou EJR (2015) [8]	Single center, Single-blinded, IV-initiated	IN.PACT, Medtronic	HPB	AVF/AVG (14/26) Exclude CVS	Clinical assessment Angiography every two months	TL primary patency at one year
Kitrou JVIR (2015) [9]	Single center, Non-blinded, IV-initiated	IN.PACT, Medtronic	HPB	AVF Exclude multi-stenosis, thrombosis	Clinical assessment Optional US	TLR-free survival period at one year
Kitrou (2017) [10]	Single center, Single-blinded, IV-initiated	Lutonix, Bard	HPB	AVF/AVG (21/19) CVS* Exclude thrombosis	Clinical assessment	Intervention-free period at 6 months
Roosen (2017) [11]	Multi-center, Single-blinded, IV-initiated	IN.PACT, Medtronic	CB	AVF/AVG (29/5) US diagnosis	US at 3, 6, 9, and 12 months	TLR-free interval
Maleux (2018) [12]	Multi-center, Non-blinded, IV-initiated	IN.PACT, Medtronic	CB	AVF Exclude CVS	Clinical assessment	Primary patency rate at 3, 6, and 12 months
Trerotola (2019) [13]	Multi-center, Single-blinded, MF-sponsored	Lutonix; Bard	CB	AVF Exclude CVS, thrombosis	Clinical assessment	TLR-free survival rate at 6 months
Björkman (2018) [14]	Single center, Single-blinded, IV-initiated	IN.PACT, Medtronic	CB	AVF Exclude CVS & thrombosis, confirmed by US & angiography	US at 1, 6, and 12 months	TLR and loss of AVF at one year
Swinnen (2018) [15]	Multi-center, Single-blinded, IV-initiated	IN.PACT, Medtronic	CB	AVF Exclude CVS & thrombosis	US baseline, 24 hours, 1 week, 6 weeks, 3, 6, and 12 months	Late lumen loss at 6 weeks, 3, 6, and 12 months
Irani (2018) [16]	Single center, Un-blinded, IV-initiated	IN.PACT, Medtronic	HPB	AVF/AVG (98/21) Exclude thrombosis	Angiography at 6 months	TL primary patency rate at 6 months
Liao (2019) [17]	Single center, Single-blinded, IV-initiated	IN.PACT, Medtronic	CB	AVG Exclude thrombosis Venous anastomotic stenosis	Clinical assessment Angiography every two months	TL primary patency rate at 6 months
Moreno-Sánchez (2020) [18]	Multi-center, Single-blinded, MF-sponsored	Passeo-18, Biotronik	HPB	AVF/AVG (136/12)^+^	Clinical assessment	TL primary patency rate at 6 and 12 months

*subclavian vein, brachiocephalic vein, superior vena cava;

^+^the number of lesions.

Abbreviations: RCTs, randomized controlled trials; DCB, drug-coated balloon; IV, investigator; HPB, high pressure balloon; AVG, arteriovenous graft; AVF, arteriovenous fistula; TL, target lesion; US, ultra-sonography; TLR, target lesion revascularization; CVS, central venous stenosis; CB, conventional balloon; MF, manufacturer.

**Table 2:** Patient characteristics and outcomes of the eligible RCTs.

Study (Year)	No. of Patients		Age	Target Lesion Primary Patency, DCB vs. SB (No. & %)			Minor and Major Complications	Follow-up Period
	DCB	CB		6 months	12 months	TLR-Free Period		
Kitrou EJR (2015) [8]	20	20	66 vs. 63	14 vs. 5 70% vs. 25%	7 vs.1 35% vs. 5%	234 vs. 131 days (median)	No	12 months
Kitrou JVIR (2015) [9]	20	20	64 vs. 57	12 vs. 6*^, ^^+^ 60% vs. 30%	5 vs. 2*^, ^^+^ 25% vs. 10%	308 vs. 161 days (median)	No	12 months
Kitrou (2017) [10]	20	18	57 vs. 57	11 vs. 5* 55% vs. 28%	3 vs. 2* 15% vs. 11%	179 vs. 124.5 days (median)	No	6 months
Roosen (2017) [11]	16	18	80 vs. 83	2 vs. 8* 13% vs. 44%	1 vs. 2* 6% vs 11%	130 vs. 189 days (mean)	1 vs. 1^++^	24 months
Maleux (2018) [12]	33	31	69 vs. 67	22 vs. 20 67% vs. 65%	14 vs. 12 42% vs. 39%	N/A	No	12 months
Trerotola (2019) [13]	141	144	64 vs. 61	87 vs. 85 70% vs. 62%	46 vs. 44 40% vs. 34%	N/A	7 vs 6^&^	24 months
Björkman (2018) [14]	18	18	67 vs. 67	4 vs. 13* 28% vs. 78%	2 vs. 14 11% vs. 78%	110 vs. 193 days (mean)	N/A	12 months
Swinnen (2018) [15]	68	60	65 vs. 65	52 vs. 28* 76% vs 47%	25 vs 14* 37% vs. 23%	42.39 vs. 10.14 months (mean)	One vessel rupture, rescued by stent graft^!^	12 months
Irani (2018) [16]	59	60	59 vs. 59	47 vs. 35 81% vs. 61%	30 vs 20^#^ 51% vs. 34%	N/A	2 vs. 1^!!^	12 months
Liao (2019) [17]	22	22	70 vs 66	9 vs. 2 41% vs. 9%	5 vs. 2 23% vs. 9%	120 vs 68 days (mean)	No	12 months
Moreno-Sánchez (2020) [18]	70^##^	78^##^	69 vs 71	57 vs. 45 73% vs. 58%	41 vs. 37 53% vs. 47%	266 vs 237 days (mean)	No major Minor, 6 vs 10	12 months

*The number was according to the number at risk of the Kaplan-Meier survival curve;

^**+**^200 days and 400 days;

^**++**^DCB group: early thrombosis of AVF (1), CB group: subtotal occlusion (1);

^&^Localized or systemic serious adverse events throughout 30 days, including periprocedural complications 2 vs 2;

^#^The number was calculated according to the patency proportion of the Kaplan-Meier survival curve;

^##^the number of lesions.

^!^Excluded from trial before randomization;

^!!^DCB group: dissection (1) and pseudoaneurysm (1); CB group: venous rupture (1)

Abbreviations: RCTs, randomized controlled trials; DCB, drug-coated balloon; CB, conventional balloon; TLR, target lesion revascularization; AVF, arteriovenous fistula; N/A, not applicable;

### Target lesion primary patency

**Fig 3:**
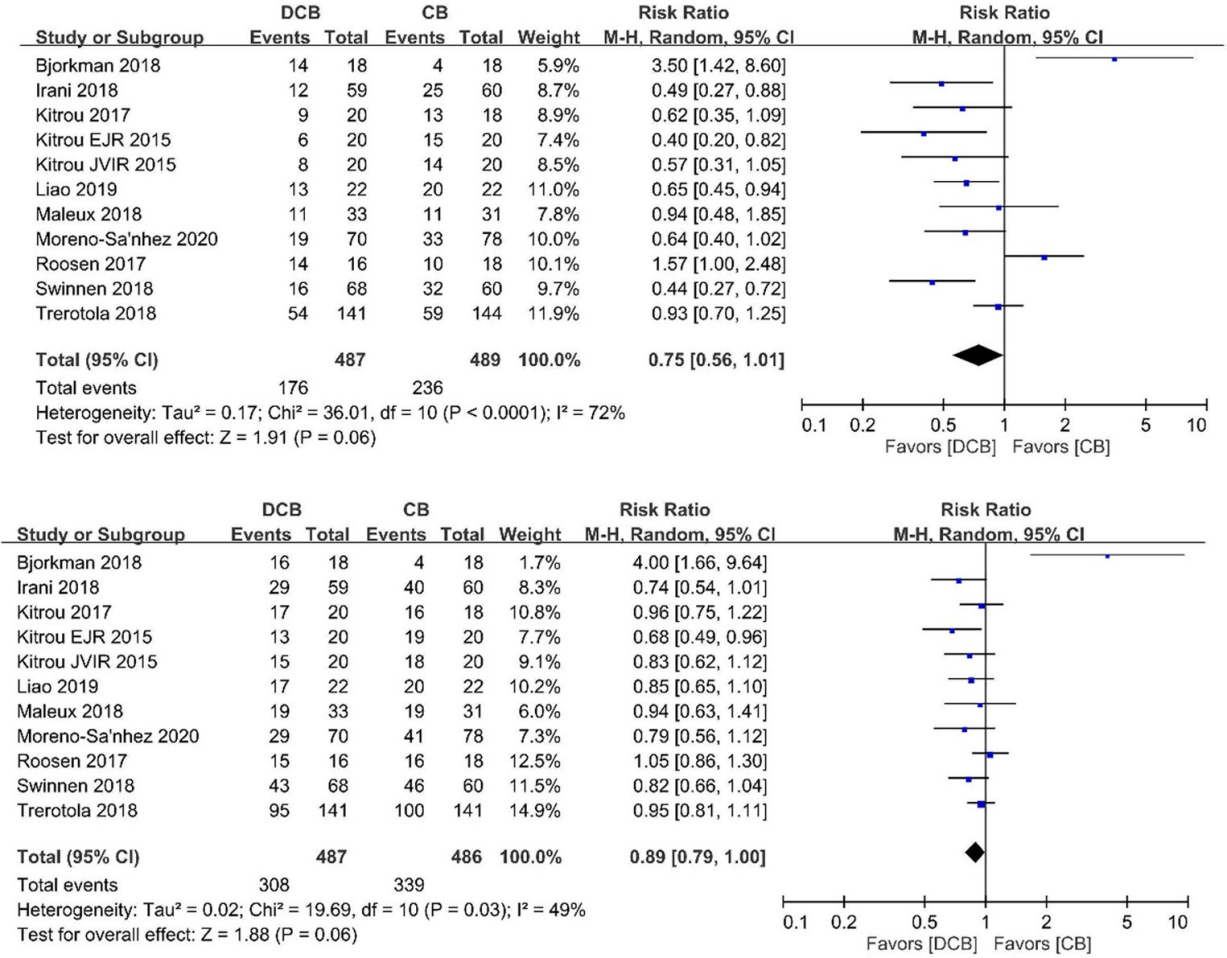
Forest plot of (A) 6-month and (B) 12-month primary patency of target lesion in DCB arm and CB arm. Abbreviations: DCB, drug-coated balloon; CB, conventional balloon;

The forest plot of the 6-month target lesion primary patency rates between DCB group and conventional group of 11 RCTs is depicted in **Fig 3A**. The event rates at 6 months in the DCB group and conventional group were 36.1% and 48.3%, respectively. Using a random-effects model, there was a non-significant decrease of 6-month event rates in the DCB group (RR 0.75; 95% CI 0.56, 1.01; *p* = 0.06, **Fig 3A**) in comparison to the conventional group.

The forest plot of the 12-month target lesion primary patency rates between DCB group and conventional group of 11 RCTs is depicted in **Fig 3B**. The event rates at 12 months in the DCB group and conventional group were 63.2% and 69.8%, respectively. Using a random-effects model, there was a non-significant decrease of 12-month event rates in the DCB group (RR 0.89; 95% CI 0.79, 1.00; *p* = 0.06, **Fig 3B**) in comparison to the CB group.

### Subgroup analysis

**Fig 4:**
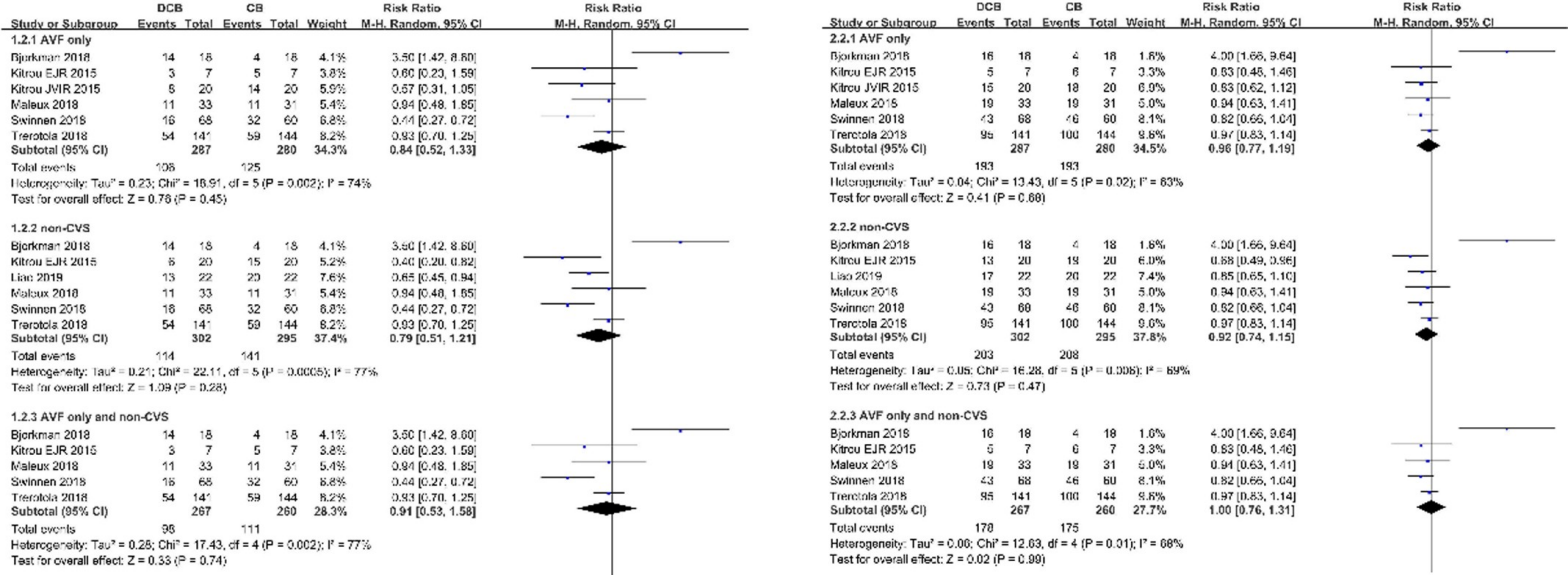
Forest plot of (A) 6-month and (B) 12-month target lesion primary patency of AVF only subgroup, non-central venous stenosis subgroup, and AVF and non-central venous stenosis subgroup. Abbreviations: DCB, drug-coated balloon; CB, conventional balloon; CVS, central venous stenosis and non-central venous stenosis and AVF only.

The combined analysis of 5 AVF-only studies and 1 AVF subgroup demonstrated similar results. The forest plot of the 6-month target lesion primary patency rates of the 6 AVF-only RCTs is depicted in **Fig 4A1**. The event rates at 6 months in the DCB group and conventional group were 36.9% and 44.6%, respectively. The decrease in 6-month event rates of DCB group was not significant compared to the conventional group (RR 0.84; 95% CI 0.52, 1.33; *p* = 0.45, **Fig 4A1**). The forest plot of the 12-month target lesion primary patency rates of the 6 AVF-only RCTs is depicted in **Fig 4B1**. The event rates at 12 months in the DCB group and conventional group were 67.2% and 68.9%, respectively. The difference in the 12-month event rates of the DCB group was not significant compared to the conventional group (RR 0.96; 95% CI 0.77, 1.19; *p* = 0.68, **Fig 4B1**).

The analysis of the 6 studies of non-central venous stenosis demonstrated similar results. The forest plot of the 6-month target lesion primary patency rates of the 6 non-central vein stenosis is depicted in **Fig 4A2**. The event rates at 6 months in the DCB group and conventional group were 37.7% and 47.8%, respectively. The decrease in 6-month event rates of DCB group was not significant compared to the conventional group (RR 0.79; 95% CI 0.51, 1.21; *p* = 0.28, **Fig 4A2**). The forest plot of the 12-month target lesion primary patency rates of the 6 non-central vein stenosis is depicted in **Fig 4B2**. The event rates at 12 months in the DCB group and conventional group were 67.2% and 70.5% respectively. The difference in 12-month event rates of DCB group was not significant compared to the conventional group (RR 0.92; 95% CI 0.74, 1.15; *p* = 0.47, **Fig 4B2**).

The subgroup analysis of the 5 studies with AVF only and non-central vein lesions demonstrated similar results. The forest plot of the 6-month target lesion primary patency rates of the 5 studies with central vein stenosis is depicted in **Fig 4A3**. The event rates at 6 months in the DCB group and conventional group were 36.7% and 42.7%, respectively. The decrease in 6-month event rates of DCB group was not significant compared to the conventional group (RR 0.91; 95% CI 0.53, 1.58; *p* = 0.74, **Fig 4A3**). The forest plot of the 12-month target lesion primary patency rates of the 5 non-central vein stenosis and AVF only is depicted in **Fig 4B3**. The event rates at 12 months in the DCB group and conventional group were 66.7% and 67.3% respectively. The difference in 12-month event rates of DCB group was not significant compared to the conventional group (RR 1.00; 95% CI 0.76, 1.31; *p* = 0.99, **Fig 4B3**).

### Access circuit primary patency

**Fig 5:**
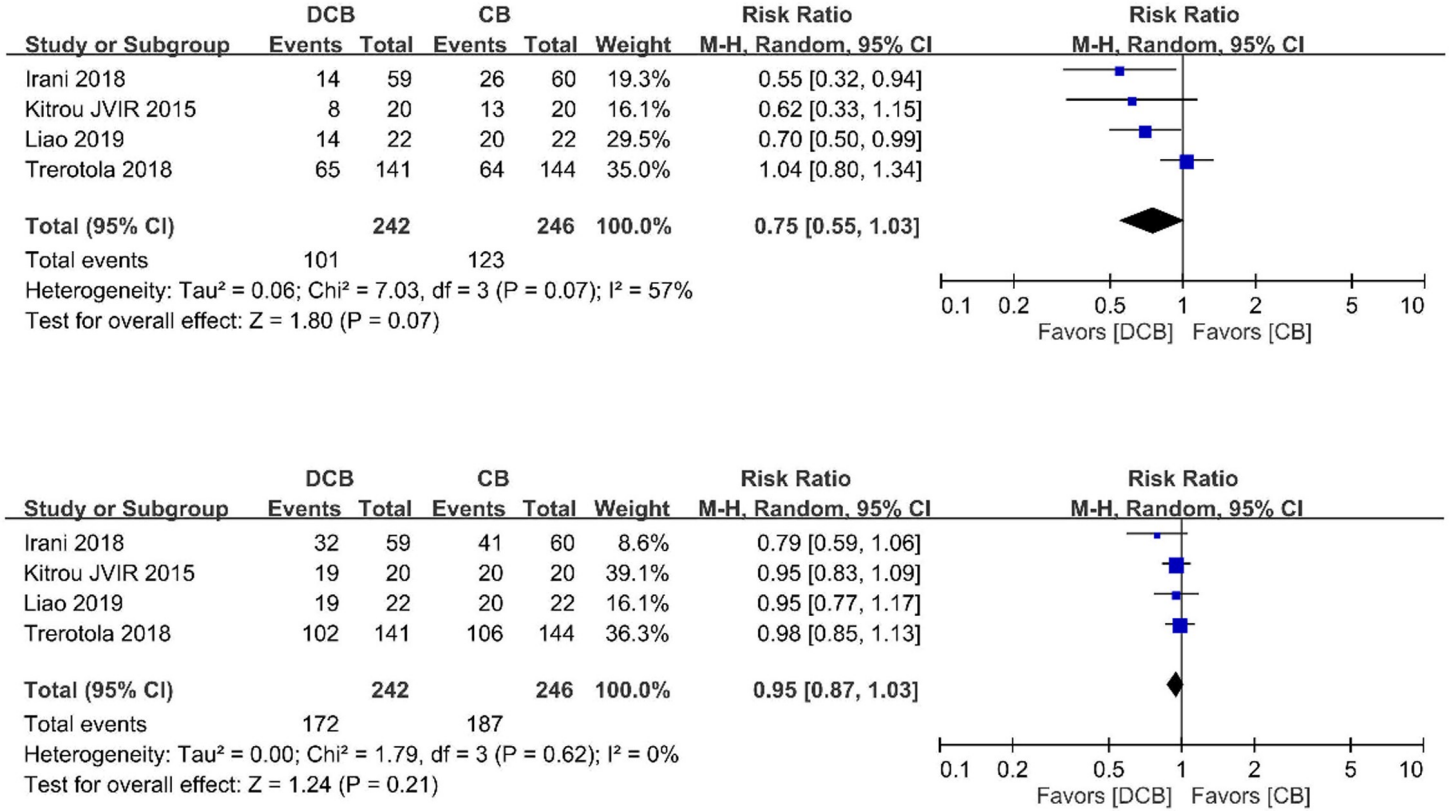
Forest plot of (A) 6-month and (B) 12-month primary patency of access circuit in the DCB arm and the CB arm. Abbreviations: DCB, drug-coated balloon; CB, conventional balloon.

The forest plot of the 6-month access circuit primary patency rates between DCB group and conventional group of 4 RCTs is depicted in **Fig 5A**. The event rates at 6 months in the DCB group and conventional group were 41.7% and 50.0%, respectively. Using a random-effects model, there was a non-significant decrease of 6-month event rates in the DCB group (RR 0.75; 95% CI 0.55, 1.03; *p* = 0.07, **Fig 5A**) in comparison to the conventional group.

The forest plot of the 12-month access circuit primary patency rates between DCB group and conventional group of 4 RCTs is depicted in **Fig 5B**. The event rates at 12 months in the DCB group and conventional group were 71.0% and 76.0%, respectively. Using a random-effects model, there was a non-significant decrease of 12-month event rates in the DCB group (RR 0.95; 95% CI 0.87, 1.03; *p* = 0.21, **Fig 5B**) in comparison to the CB group.

### Analysis of procedure-related complications

**Fig 6:**
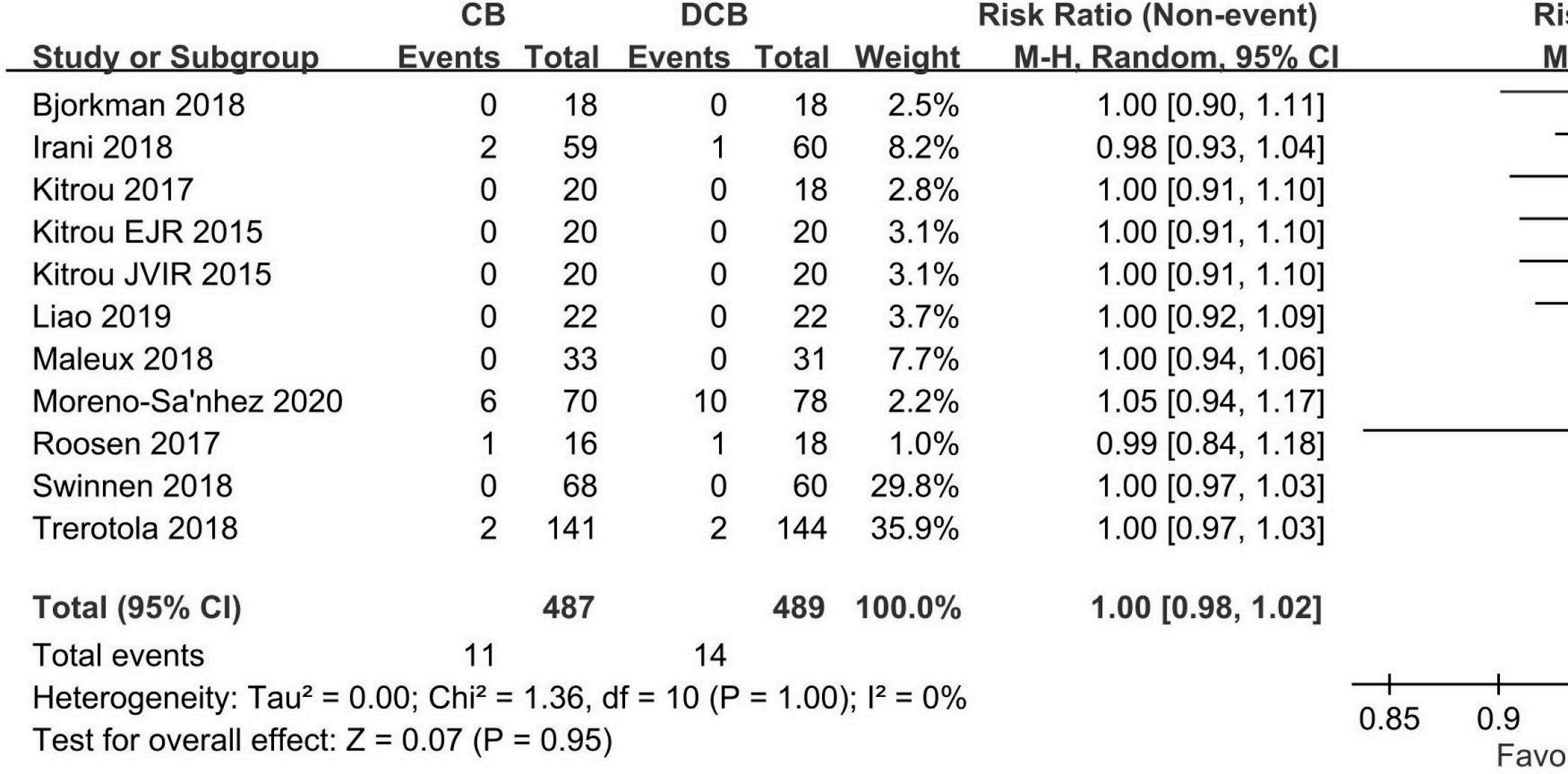
Forest plot of complications in the DCB arm and CB arm. Abbreviations: DCB, drug-coated balloon; CB, conventional balloon;

The procedure-related complication rate was rare and there was no significant difference between the two groups (RR 1.00; 95% CI (0.98, 1.02); *p* = 0.95, **Fig 6**).

### Publication bias and heterogeneity

**Fig 7:**
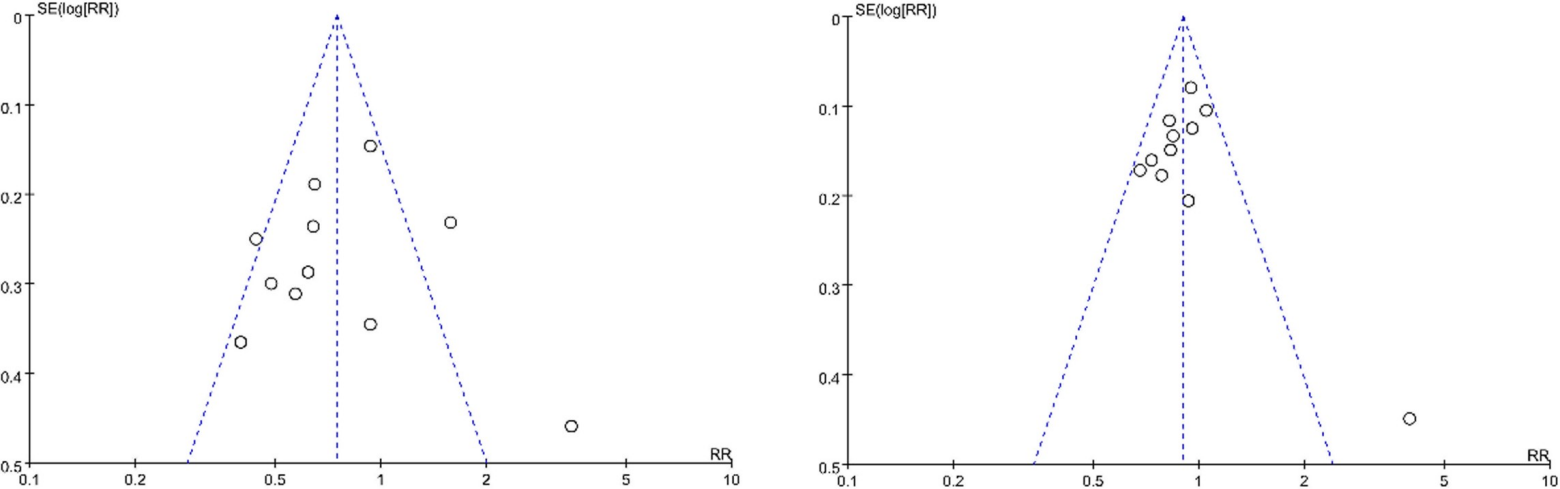
Funnel plot of publication bias. A. 6-month primary patency B. 12-month primary patency.

Apparent publication bias was detected by visually assessing the funnel plot of the 6-month target lesion primary patency rates in **Fig 7A** and the 12-month target lesion primary patency rates in **Fig 7B**. Statistical analyses for both 6-month primary patency (I^2^ = 72%; *p* < 0.0001, **Fig 3A**) and 12-month primary patency (I^2^ = 49%; *p* = 0.03, **Fig 3B**) showed intermediate heterogeneity.

## Discussion

### Main findings

This meta-analysis of RCTs demonstrated modest numeric improvements of the target lesion primary patency rate of DCB angioplasty, but the differences were not statistically significant. A wide heterogeneity of patency outcomes among studies was observed. There was no significant difference observed in the target lesion patency between the DCB and CB angioplasty, even with the subgroup analysis of the AVF-only or non-central venous stenosis.

### Comparison to previous studies

Our conclusions are different from the 2 meta-analysis studies recently published [19, 20]. Kennedy et al. reported a significant improvement of patency in the DCB group, both for patency at 6 months (OR 0.40; CI 0.23–0.70) and 12 months (OR 0.20; CI 0.07–0.62). Wee et al. also reported a better patency outcome for DCB angioplasty, both for the patency at 6 months (RR 0.57; CI 0.63–0.84) and 12 months (RR 0.73; CI 0.63–0.84).

There were differences in the methodology responsible for the disparity among meta-analysis studies on the same issue. First, only randomized control studies were used for the meta-analysis conducted in our study; the other studies included cohort studies and retrospective studies that were inherent to selection bias. For example, Kennedy’s study [19] included a retrospective study [21], a prospective study [22], and a prospective cohort [23]. Second, the number of censored cases during follow-up was not provided in some of the studies. These cases were counted as the proportion without events in Kennedy’s study, which obviously underestimated the number of events that occurred. In contrast, the number of events were derived from the proportions of the survival curve or the text in our analysis and Wee’s analysis [20]. The derived number of events might then overestimate the true number. We took a more rigorous manner to treat these early-censored cases. This approach represents a more conservative attitude on the evaluation of a new device. Third, we performed a more comprehensive search for RCTs and adhered to the results of the quality assessment. In contrast, two RCTs with neutral or opposite results were not included in Kennedy’s and Wee’s analysis. A sensitivity test by excluding these two trials showed a modest but significant benefit favoring DCB [10, 14]. Nonetheless, these two studies were reserved in our formal analysis because they fulfilled the prespecified quality assessment. Finally, in the AVF subgroup analysis, some studies that enrolled patients with either AVFs or AVGs were included in the meta-analysis by Kennedy et al. [11, 16].

### Heterogeneity

Our meta-analysis demonstrated an intermediate heterogeneity of patency outcomes across studies (6-month patency I^2^, 72%; 12-month patency I^2^, 49%). A variety of vascular access factors may contribute to the variation. First, the distribution of anatomical stenosis varied widely among these studies. The pathophysiology of stenosis depends on the biomechanical properties which may differ among the anatomical sites. The response to DCB angioplasty among different sites, such as anastomosis, graft junctions, cephalic arch, or central veins, might be quite different. Second, AVF was the predominant vascular access in most studies, but there were 4 studies that enrolled patients with either AVFs or AVGs. The AVF patency post-angioplasty was usually superior to that of AVG patency [24]. An early study by Kitrou et al. showed good DCB results mainly in AVGs but not in AVFs [9]. Third, 1 study enrolled immature or young AVFs created for less than one year [14]. In contrast, other studies enrolled vascular accesses aged 2.5 to 3.5 years. The study of young AVFs showed inferior results with DCB angioplasty, raising the concern on safety of DCB within thin venous walls. Fourth, restenotic lesions comprised majority of the cases, but 4 of these studies enrolled both recurrent and de novo lesions. Restenotic lesions are usually prone to frequent re-interventions. Irani et al. also demonstrated that DCB angioplasty offered a greater benefit for restenotic lesions than de novo lesions [16].

Variations in devices, techniques, and study methods among studies also contribute to the heterogeneity. DCB technology usually consisted of a normal-pressure balloon that achieves uniform drug coating and delivery to vessel wall. Nowadays, pre-dilatation to facilitate drug diffusion within the deeper layer of the vessel wall is recommended by the manufacturers. In earlier studies, pre-dilatation was not performed routinely before the application of DCBs [8]. The inflation times ranged from 1 to 3 minutes among the studies. The paclitaxel dosage on angioplasty balloons were also different among the DCBs used (3.5 ug/mm^2^ for the InPact Admiral balloon catheter; 2.0 ug/mm^2^ for the Lutonix balloon). However, the optimal dosage and DCB technology for hemodialysis vascular access was not clear because pre-clinical or experimental studies in animal models are missing. Variations between studies were also noted for the inclusion and exclusion criteria and surveillance protocol after angioplasty [8–10, 12, 13]. All the aforementioned factors could contribute to the heterogeneity in our meta-analysis.

### Subgroup analysis

Because of the high heterogeneity, subgroup analysis was performed to address the interference of lesion characteristics (central vein) and access factors (AVF). The subgroup analysis of AVF cases from 6 RCTs demonstrated no significant improvement in target lesion primary patency, either at 6 months or at 12 months. The heterogeneity across studies remained high after excluding studies that also enrolled patients with AVGs. Most studies did not report the results of AVFs and AVGs separately, let alone a subgroup analysis for AVGs. In the subgroup analysis of studies excluding central vein lesions, DCB still failed to show superiority over CB, either at the 6-month or 12-month follow-up period. The heterogeneity remained high across studies. Despite our attempt to stratify these data, DCB still failed to show patency benefits over CB. Nonetheless, a variety of important subgroups could not be accounted for because of the deficiency of information in the original publications. An international collaboration to share patient-level data may help to clarify the effect of DCB on specific subgroups.

### Safety

No difference in procedural-related complications between DCB and conventional angioplasty was found. Because of the rarity of complications, the analysis was underpowered to detect a significant difference in complications. Besides, some relevant complications, such as thrombosis of vascular accesses during follow-up, were not specified in the majority of studies. A recent meta-analysis found an increased mortality risk at 2 and 5 years after the application of paclitaxel-coated devices on patients with femoropopliteal artery diseases [25]. While there is no plausible biological mechanism, it raised a concern around drug toxicity. Patients who suffer from ESRD have a high risk of death and also a high chance of repeated interventions. Regarding DCB angioplasty of hemodialysis vascular access, a recent meta-analysis showed that there was no increase in mortality rate for up to 2 years [26]. Further studies are needed to investigate the long-term safety within this high-risk population.

### Why are outcomes different from arterial angioplasty?

A variety of factors may be responsible for DCB being less effective in hemodialysis vascular access. First, DCB technology was based on animal models on atherosclerotic lesions. Information demonstrating the diffusion and duration of paclitaxel within the venous wall was not available. Second, hemodynamic shear stress, repeat needle injury, and uremia were persistently elevated in hemodialysis vascular access [27]. Nonetheless, the effect of paclitaxel persisted only for a limited period in the vessel wall. Third, the pathology of vascular access stenosis is predominantly neointimal hyperplasia, which is very different from atherosclerosis [28, 29]. Fourth, veins contain less elastic lamina than arteries, which may predispose the migration of smooth muscle cells into neointima [29].

### Limitations

There are some limitations encountered that should be addressed. First, further analysis based on lesion types might be helpful to clarify the heterogenicity of outcomes among these studies. Nonetheless, this is not possible because patient-level data were not available, despite our efforts to contact the authors of the included studies. Second, the numbers of events were derived from the proportions displayed on the graph or the context in some studies if the number of censored observations were not available. The derivation may overestimate the numbers of events. Thirdly, cost-effectiveness is a critical issue for the application of such an expensive device, but it was not addressed in this study. A long-term cost-effectiveness analysis is needed to clarify the value of DCB in clinical practice. Finally, there are studies of DCB on hemodialysis vascular accesses ongoing and the results of meta-analysis might be different as these studies published.

## Conclusion

Our results demonstrated that DCB angioplasty showed a modest but non-significant decrease of restenosis compared to conventional angioplasty in the treatment of hemodialysis vascular access dysfunction. A wide heterogeneity across the studies was present, even in the subgroup analysis. Future studies focused on a specific type of vascular access or on the characteristics of lesions, under optimized techniques of application, are warranted to clarify the role of DCB in hemodialysis vascular access management.

## Supporting information

S1 File Appendix 1 search strategy (DOCX)

S1 Data (PDF)
