# Correction: Drug-coated balloon versus conventional balloon angioplasty of hemodialysis arteriovenous fistula or graft: A systematic review and meta-analysis of randomized controlled trials

**DOI:** 10.1371/journal.pone.0233923

**Published:** 2020-05-22

**Authors:** The PLOS ONE Staff

In Fig 6, the right column is truncated. Please see the correct Fig 6 here. The publisher apologizes for the error.

**Fig 6:**
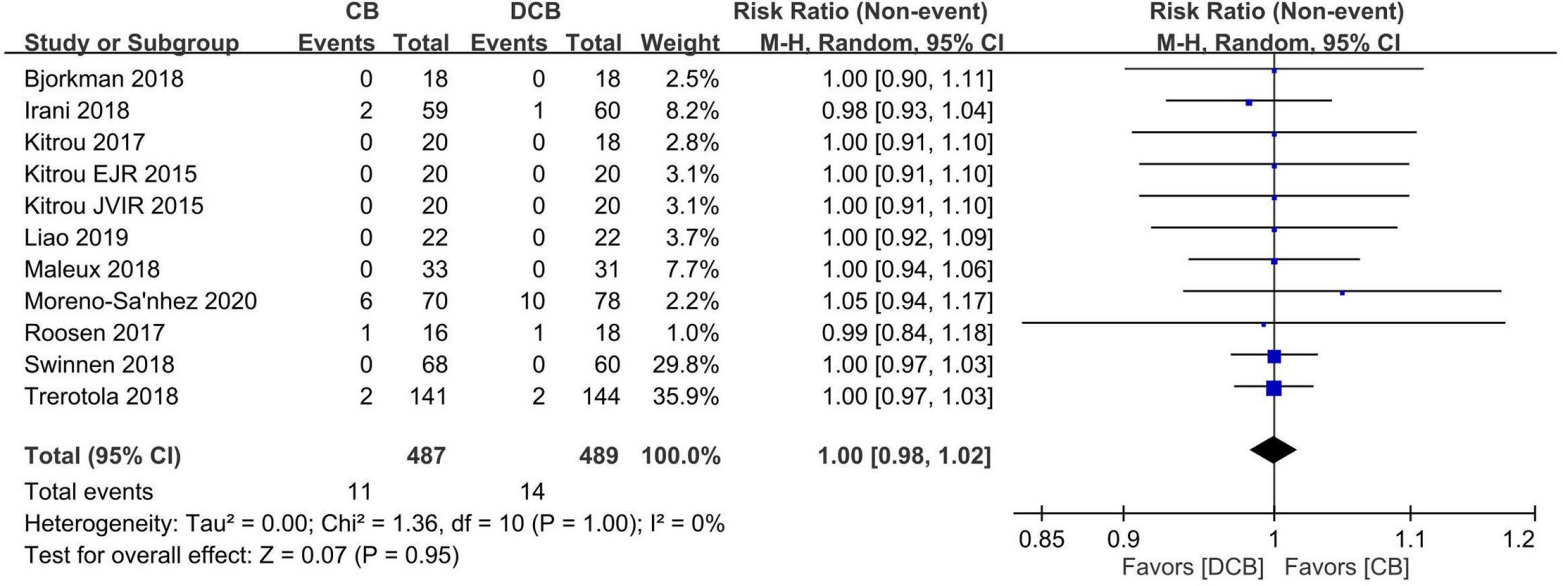
Forest plot of complications in the DCB arm and CB arm. Abbreviations: DCB, drug-coated balloon; CB, conventional balloon.
