# Supplementary material for: Drug-coated balloon versus conventional balloon angioplasty of hemodialysis arteriovenous fistula or graft: A systematic review and meta-analysis of randomized controlled trials
Source: PLoS One. 2020 Apr 14;15(4):e0231463. doi: 10.1371/journal.pone.0231463 (PMC7156061; doi:10.1371/journal.pone.0231463)
Supplement: S1 File — (DOCX) [file pone.0231463.s001.docx]

Pubmed (689), all fields (2020-03-09)

(((((((((Arteriovenous graft) OR Arteriovenous fistula) OR Arteriovenous shunt) OR Vascular access) OR Dialysis access) OR Arteriovenous Anastomosis) OR Arteriovenous Shunt, Surgical) OR Blood Vessel Prosthesis)) AND ((((Drug eluting balloon) OR Paclitaxel coated balloon) OR Drug coated balloon) OR paclitaxel)

Embase (372), all fields (2020-03-09)

#1 ‘Arteriovenous graft’

#2 ‘Arteriovenous fistula’

#3 ‘Arteriovenous shunt’

#4 ‘Vascular access’

#5 ‘Dialysis access’

#6 ‘Arteriovenous Anastomosis’

#7 ‘Arteriovenous Shunt, Surgical’

#8 ‘Blood Vessel Prosthesis’

#9 #1 OR #2 OR #3 OR #4 OR #5 OR #6 OR #7 OR #8

#10 ’Drug eluting balloon’

#11 ‘Paclitaxel coated balloon’

#12 ‘Drug coated balloon’

#13 ‘paclitaxel’

#14 #10 OR #11 OR #12 OR #13

#15 #9 AND #14

Cochrane library (149), Title, Abstract, Keyword (2020-03-09)

#1 “Arteriovenous graft”

#2 “Arteriovenous fistula”

#3 “Arteriovenous shunt”

#4 “Vascular access”

#5 “Dialysis access”

#6 “Arteriovenous Anastomosis”

#7 “Arteriovenous Shunt, Surgical”

#8 “Blood Vessel Prosthesis”

#9 #1 OR #2 OR #3 OR #4 OR #5 OR #6 OR #7 OR #8

#10 ”Drug eluting balloon”

#11 “Paclitaxel coated balloon”

#12 “Drug coated balloon”

#13 “paclitaxel”

#14 #10 OR #11 OR #12 OR #13 OR #14

#15 #9 AND #14
